# Supplementary material for: Comparative genomics provides new insights into the diversity, physiology, and sexuality of the only industrially exploited tremellomycete: Phaffia rhodozyma
Source: BMC Genomics. 2016 Nov 9;17:901. doi: 10.1186/s12864-016-3244-7 (PMC5103461; doi:10.1186/s12864-016-3244-7)
Supplement: Additional file 6: — List of orphan genes with links to PFAM (related to Additional file 1: Table S1). (ZIP 1428 kb) [file 12864_2016_3244_MOESM6_ESM.zip › BLAST_HTML_FTR/G05849_P.html]

BLAST Search Results


```
BLASTP 2.2.27+


Reference:
Stephen F. Altschul, Thomas L. Madden, Alejandro A. Schäffer,
Jinghui Zhang, Zheng Zhang, Webb Miller, and David J. Lipman (1997),
"Gapped BLAST and PSI-BLAST: a new generation of protein database
search programs", Nucleic Acids Res. 25:3389-3402.


Reference for
composition-based statistics:
Alejandro A. Schäffer, L. Aravind, Thomas L. Madden, Sergei
Shavirin, John L. Spouge, Yuri I. Wolf, Eugene V. Koonin, and
Stephen F. Altschul (2001), "Improving the accuracy of PSI-BLAST
protein database searches with composition-based statistics and
other refinements", Nucleic Acids Res. 29:2994-3005.


Database: nr
           71,551,133 sequences; 26,053,659,533 total letters


Query= G05849_P

Length=103
                                                                      Score     E
Sequences producing significant alignments:                          (Bits)  Value

gb|AHZ31749.1|  chalcone isomerase [Viola x wittrockiana]             38.5    0.38 
ref|WP_004177175.1|  glutamate--ammonia-ligase adenylyltransferas...  38.9    0.48 
ref|WP_041512846.1|  glutamine-synthetase adenylyltransferase [Ni...  37.4    1.6  
gb|KID82326.1|  hypothetical protein MGU_10356 [Metarhizium guizh...  36.6    2.6  
ref|WP_033240455.1|  histidine kinase [Streptomyces griseus]          36.6    2.9  
ref|WP_052861123.1|  histidine kinase [Streptomyces celluloflavus]    36.2    3.2  
gb|KON27826.1|  hypothetical protein AC474_02765 [Marine benthic ...  36.2    3.3  
ref|WP_044367740.1|  histidine kinase [Streptomyces natalensis] >...  36.2    3.3  
gb|KPC59434.1|  histidine kinase [Streptomyces chattanoogensis]       36.2    3.3  
ref|WP_018957051.1|  histidine kinase [Streptomyces sp. CNB091]       36.2    3.3  
ref|WP_046925558.1|  histidine kinase [Streptomyces lydicus] >gb|...  36.2    3.4  
ref|WP_030021236.1|  histidine kinase [Streptomyces monomycini]       36.2    3.4  
ref|WP_031011508.1|  histidine kinase [Streptomyces sp. NRRL WC-3...  36.2    3.4  
gb|EPD65643.1|  hypothetical protein HMPREF1211_02198 [Streptomyc...  36.2    3.5  
ref|WP_037781056.1|  histidine kinase [Streptomyces sp. HGB0020]      36.2    3.5  
ref|WP_003965724.1|  MULTISPECIES: histidine kinase [Streptomyces...  36.2    3.6  
ref|WP_016570935.1|  MULTISPECIES: two-component system sensor ki...  36.2    4.0  
ref|WP_032777362.1|  histidine kinase, partial [Streptomyces albus]   35.8    4.6  
ref|WP_041131654.1|  histidine kinase [Streptomyces vietnamensis]...  35.8    4.9  
ref|WP_033204353.1|  MULTISPECIES: histidine kinase [Streptomyces...  35.8    5.1  
ref|WP_037642790.1|  histidine kinase, partial [Streptomyces bico...  35.8    5.1  
ref|WP_017237682.1|  histidine kinase [Streptomyces sp. SS]           35.8    5.2  
ref|WP_026251764.1|  histidine kinase [Streptomyces sp. BoleA5]       35.8    5.2  
ref|WP_030211244.1|  histidine kinase [Streptomyces bikiniensis]      35.8    5.3  
ref|WP_030623816.1|  MULTISPECIES: histidine kinase [Streptomyces]    35.4    5.8  
ref|WP_028441142.1|  MULTISPECIES: histidine kinase [Streptomyces...  35.4    5.8  
ref|WP_014055421.1|  histidine kinase [Streptomyces violaceusnige...  35.4    6.4  
ref|WP_003955552.1|  histidine kinase [Streptomyces clavuligerus]...  35.0    8.5  
ref|XP_002980315.1|  hypothetical protein SELMODRAFT_444519 [Sela...  35.0    9.3  
ref|WP_038465608.1|  hypothetical protein [Candidatus Paracaediba...  35.0    9.4  


 >gb|AHZ31749.1| chalcone isomerase [Viola x wittrockiana]
Length=216

 Score = 38.5 bits (88),  Expect = 0.38, Method: Compositional matrix adjust.
 Identities = 24/72 (33%), Positives = 37/72 (51%), Gaps = 4/72 (6%)

Query  22   TRNHFKKEKNEECIAKTKEVFEEYGIGRDSVDHTVNVFRHLVEEELYALLASDLSTDNPL  81
            T + + ++ +E C+A  K V    GI  D+    +  FR + +EE +   +S L T  PL
Sbjct  104  TGHQYSEKVSENCVAYWKSV----GIYTDAESKAIEEFRQVFKEETFPPGSSILFTQTPL  159

Query  82   ATLSSSVSSTGS  93
             TL+ S S  GS
Sbjct  160  GTLAISFSKDGS  171


>ref|WP_004177175.1| glutamate--ammonia-ligase adenylyltransferase [Nitrosospira lacus]
 emb|CCU62324.1| Glutamate-ammonia-ligase adenylyltransferase [Nitrosospira lacus]
Length=933

 Score = 38.9 bits (89),  Expect = 0.48, Method: Composition-based stats.
 Identities = 19/42 (45%), Positives = 26/42 (62%), Gaps = 0/42 (0%)

Query  48   GRDSVDHTVNVFRHLVEEELYALLASDLSTDNPLATLSSSVS  89
            GRD+V+  ++V RH    E++ LLA DL    PL TLS  +S
Sbjct  590  GRDNVEQQMDVLRHFHHTEVFRLLARDLEGLLPLETLSDHLS  631


>ref|WP_041512846.1| glutamine-synthetase adenylyltransferase [Nitrosospira sp. NpAV]
 gb|KIO50089.1| glutamine-synthetase adenylyltransferase [Nitrosospira sp. NpAV]
Length=930

 Score = 37.4 bits (85),  Expect = 1.6, Method: Composition-based stats.
 Identities = 17/44 (39%), Positives = 26/44 (59%), Gaps = 0/44 (0%)

Query  46   GIGRDSVDHTVNVFRHLVEEELYALLASDLSTDNPLATLSSSVS  89
            G GR+ ++  ++V RH    E++ LLA D+    PL TLS  +S
Sbjct  588  GFGREDIEQQMDVLRHFHHAEVFRLLARDVEGLLPLETLSDHLS  631


>gb|KID82326.1| hypothetical protein MGU_10356 [Metarhizium guizhouense ARSEF 
977]
Length=350

 Score = 36.6 bits (83),  Expect = 2.6, Method: Composition-based stats.
 Identities = 19/49 (39%), Positives = 27/49 (55%), Gaps = 3/49 (6%)

Query  50   DSVDHTVNVFRHLVEEELYALLASDLSTDNPLATLSSSVSSTGSDDDAW  98
            D  DH V V +++ ++  YALL   L  DNP+   S+SV+    D  AW
Sbjct  98   DGEDHCVEVVKNIKKQPAYALL---LGQDNPICLASASVTYPSGDRYAW  143


>ref|WP_033240455.1| histidine kinase [Streptomyces griseus]
Length=852

 Score = 36.6 bits (83),  Expect = 2.9, Method: Composition-based stats.
 Identities = 19/39 (49%), Positives = 26/39 (67%), Gaps = 2/39 (5%)

Query  63   VEEELYALLASDLSTDNPLATLSSSVSSTGSDDDAWSEE  101
            +   L A ++ DL T  PLAT+ +SV+S  SDD AWSE+
Sbjct  618  IRTALLAAVSHDLRT--PLATIKASVTSLRSDDVAWSEQ  654


>ref|WP_052861123.1| histidine kinase [Streptomyces celluloflavus]
Length=850

 Score = 36.2 bits (82),  Expect = 3.2, Method: Composition-based stats.
 Identities = 19/39 (49%), Positives = 26/39 (67%), Gaps = 2/39 (5%)

Query  63   VEEELYALLASDLSTDNPLATLSSSVSSTGSDDDAWSEE  101
            +   L A ++ DL T  PLA++ +SV+S  SDD AWSEE
Sbjct  617  IRTALLAAVSHDLRT--PLASIKASVTSLRSDDVAWSEE  653


>gb|KON27826.1| hypothetical protein AC474_02765 [Marine benthic group B archaeon 
SG8-8-1]
Length=693

 Score = 36.2 bits (82),  Expect = 3.3, Method: Composition-based stats.
 Identities = 28/101 (28%), Positives = 49/101 (49%), Gaps = 7/101 (7%)

Query  6    PEPSVPLKAILKKKTNTRNHFKKEKNEECIAKTKEVFEEYGIGRDSVDHTVNVFRHL-VE  64
            P   +P+K +L K+ N    F+   + +    +KE+F + G  R+    T  +F HL   
Sbjct  33   PNVIIPIKNVLMKQFNFIQEFE---DHDLFIISKEIFLKIGFLREKFKDTGFIFSHLGTL  89

Query  65   EELYALLASDL---STDNPLATLSSSVSSTGSDDDAWSEEL  102
            E+  ++L  +L   S DN L+ +  ++ ST  D D    E+
Sbjct  90   EKFESILQKNLEIFSKDNVLSIIPFNIPSTAIDKDFADREI  130


>ref|WP_044367740.1| histidine kinase [Streptomyces natalensis]
 gb|KIZ14616.1| histidine kinase [Streptomyces natalensis ATCC 27448]
Length=850

 Score = 36.2 bits (82),  Expect = 3.3, Method: Composition-based stats.
 Identities = 19/39 (49%), Positives = 26/39 (67%), Gaps = 2/39 (5%)

Query  63   VEEELYALLASDLSTDNPLATLSSSVSSTGSDDDAWSEE  101
            +   L A ++ DL T  PLA++ +SV+S  SDD AWSEE
Sbjct  617  IRTALLAAVSHDLRT--PLASIKASVTSLRSDDVAWSEE  653


>gb|KPC59434.1| histidine kinase [Streptomyces chattanoogensis]
Length=851

 Score = 36.2 bits (82),  Expect = 3.3, Method: Composition-based stats.
 Identities = 19/39 (49%), Positives = 26/39 (67%), Gaps = 2/39 (5%)

Query  63   VEEELYALLASDLSTDNPLATLSSSVSSTGSDDDAWSEE  101
            +   L A ++ DL T  PLA++ +SV+S  SDD AWSEE
Sbjct  618  IRTALLAAVSHDLRT--PLASIKASVTSLRSDDVAWSEE  654


>ref|WP_018957051.1| histidine kinase [Streptomyces sp. CNB091]
Length=847

 Score = 36.2 bits (82),  Expect = 3.3, Method: Composition-based stats.
 Identities = 19/39 (49%), Positives = 26/39 (67%), Gaps = 2/39 (5%)

Query  63   VEEELYALLASDLSTDNPLATLSSSVSSTGSDDDAWSEE  101
            +   L A ++ DL T  PLA++ ++VSS  SDD AWSEE
Sbjct  614  IRTALLAAVSHDLRT--PLASIKAAVSSLRSDDVAWSEE  650


>ref|WP_046925558.1| histidine kinase [Streptomyces lydicus]
 gb|AJT63838.1| two-component system sensor kinase [Streptomyces lydicus A02]
Length=851

 Score = 36.2 bits (82),  Expect = 3.4, Method: Composition-based stats.
 Identities = 19/39 (49%), Positives = 26/39 (67%), Gaps = 2/39 (5%)

Query  63   VEEELYALLASDLSTDNPLATLSSSVSSTGSDDDAWSEE  101
            +   L A ++ DL T  PLA++ +SV+S  SDD AWSEE
Sbjct  618  IRTALLAAVSHDLRT--PLASIKASVTSLRSDDVAWSEE  654


>ref|WP_030021236.1| histidine kinase [Streptomyces monomycini]
Length=855

 Score = 36.2 bits (82),  Expect = 3.4, Method: Composition-based stats.
 Identities = 19/39 (49%), Positives = 26/39 (67%), Gaps = 2/39 (5%)

Query  63   VEEELYALLASDLSTDNPLATLSSSVSSTGSDDDAWSEE  101
            +   L A ++ DL T  PLA++ +SV+S  SDD AWSEE
Sbjct  622  IRTALLAAVSHDLRT--PLASIKASVTSLRSDDVAWSEE  658


>ref|WP_031011508.1| histidine kinase [Streptomyces sp. NRRL WC-3773]
Length=855

 Score = 36.2 bits (82),  Expect = 3.4, Method: Composition-based stats.
 Identities = 19/39 (49%), Positives = 26/39 (67%), Gaps = 2/39 (5%)

Query  63   VEEELYALLASDLSTDNPLATLSSSVSSTGSDDDAWSEE  101
            +   L A ++ DL T  PLA++ +SV+S  SDD AWSEE
Sbjct  622  IRTALLAAVSHDLRT--PLASIKASVTSLRSDDVAWSEE  658


>gb|EPD65643.1| hypothetical protein HMPREF1211_02198 [Streptomyces sp. HGB0020]
Length=852

 Score = 36.2 bits (82),  Expect = 3.5, Method: Composition-based stats.
 Identities = 20/39 (51%), Positives = 25/39 (64%), Gaps = 2/39 (5%)

Query  63   VEEELYALLASDLSTDNPLATLSSSVSSTGSDDDAWSEE  101
            +   L A ++ DL T  PLA + +SVSS  SDD AWSEE
Sbjct  616  IRTALLAAVSHDLRT--PLAGIKASVSSLRSDDVAWSEE  652


>ref|WP_037781056.1| histidine kinase [Streptomyces sp. HGB0020]
Length=849

 Score = 36.2 bits (82),  Expect = 3.5, Method: Composition-based stats.
 Identities = 20/39 (51%), Positives = 25/39 (64%), Gaps = 2/39 (5%)

Query  63   VEEELYALLASDLSTDNPLATLSSSVSSTGSDDDAWSEE  101
            +   L A ++ DL T  PLA + +SVSS  SDD AWSEE
Sbjct  613  IRTALLAAVSHDLRT--PLAGIKASVSSLRSDDVAWSEE  649


>ref|WP_003965724.1| MULTISPECIES: histidine kinase [Streptomyces]
 dbj|BAG18490.1| putative two-component system sensor kinase [Streptomyces griseus 
subsp. griseus NBRC 13350]
 gb|EGE41270.1| Osmosensitive K channel His kinase sensor [Streptomyces sp. ACT-1]
Length=847

 Score = 36.2 bits (82),  Expect = 3.6, Method: Composition-based stats.
 Identities = 19/39 (49%), Positives = 26/39 (67%), Gaps = 2/39 (5%)

Query  63   VEEELYALLASDLSTDNPLATLSSSVSSTGSDDDAWSEE  101
            +   L A ++ DL T  PLA++ ++VSS  SDD AWSEE
Sbjct  614  IRTALLAAVSHDLRT--PLASIKAAVSSLRSDDVAWSEE  650


>ref|WP_016570935.1| MULTISPECIES: two-component system sensor kinase [Streptomyces]
 gb|EOT05432.1| histidine kinase [Streptomyces albulus CCRC 11814]
 gb|EXU86387.1| histidine kinase [Streptomyces albulus PD-1]
 gb|AIA06394.1| two-component system sensor kinase for high-affinity potassium 
transport system [Streptomyces albulus]
 gb|AKA03097.1| histidine kinase [Streptomyces albulus ZPM]
Length=851

 Score = 36.2 bits (82),  Expect = 4.0, Method: Composition-based stats.
 Identities = 19/39 (49%), Positives = 26/39 (67%), Gaps = 2/39 (5%)

Query  63   VEEELYALLASDLSTDNPLATLSSSVSSTGSDDDAWSEE  101
            +   L A ++ DL T  PLA++ +SV+S  SDD AWSEE
Sbjct  618  IRTALLAAVSHDLRT--PLASIKASVTSLRSDDVAWSEE  654


>ref|WP_032777362.1| histidine kinase, partial [Streptomyces albus]
Length=695

 Score = 35.8 bits (81),  Expect = 4.6, Method: Composition-based stats.
 Identities = 19/39 (49%), Positives = 25/39 (64%), Gaps = 2/39 (5%)

Query  63   VEEELYALLASDLSTDNPLATLSSSVSSTGSDDDAWSEE  101
            +   L A ++ DL T  PLA + ++VSS  SDD AWSEE
Sbjct  462  IRTALLAAVSHDLRT--PLAAIKAAVSSLRSDDVAWSEE  498


>ref|WP_041131654.1| histidine kinase [Streptomyces vietnamensis]
 gb|AJF67723.1| histidine kinase [Streptomyces vietnamensis]
Length=849

 Score = 35.8 bits (81),  Expect = 4.9, Method: Composition-based stats.
 Identities = 19/39 (49%), Positives = 25/39 (64%), Gaps = 2/39 (5%)

Query  63   VEEELYALLASDLSTDNPLATLSSSVSSTGSDDDAWSEE  101
            +   L A ++ DL T  PLA++ +SVSS  SDD  WSEE
Sbjct  614  IRTSLLAAVSHDLRT--PLASIKASVSSLRSDDVEWSEE  650


>ref|WP_033204353.1| MULTISPECIES: histidine kinase [Streptomyces]
 gb|KOG33819.1| histidine kinase [Streptomyces viridochromogenes]
Length=848

 Score = 35.8 bits (81),  Expect = 5.1, Method: Composition-based stats.
 Identities = 19/39 (49%), Positives = 25/39 (64%), Gaps = 2/39 (5%)

Query  63   VEEELYALLASDLSTDNPLATLSSSVSSTGSDDDAWSEE  101
            +   L A ++ DL T  PLA++ +SVSS  SDD  WSEE
Sbjct  614  IRTSLLAAVSHDLRT--PLASIKASVSSLRSDDVEWSEE  650


>ref|WP_037642790.1| histidine kinase, partial [Streptomyces bicolor]
Length=690

 Score = 35.8 bits (81),  Expect = 5.1, Method: Composition-based stats.
 Identities = 24/60 (40%), Positives = 32/60 (53%), Gaps = 7/60 (12%)

Query  47   IGRDSVDHTVNVFRHLVE-----EELYALLASDLSTDNPLATLSSSVSSTGSDDDAWSEE  101
            + R  + H  +  + L E       L A ++ DL T  PLA + +SVSS  SDD AWSEE
Sbjct  375  LDRQRLRHEADQAKELAEGNRIRTALLAAVSHDLRT--PLAGIKASVSSLRSDDVAWSEE  432


>ref|WP_017237682.1| histidine kinase [Streptomyces sp. SS]
Length=848

 Score = 35.8 bits (81),  Expect = 5.2, Method: Composition-based stats.
 Identities = 19/39 (49%), Positives = 25/39 (64%), Gaps = 2/39 (5%)

Query  63   VEEELYALLASDLSTDNPLATLSSSVSSTGSDDDAWSEE  101
            +   L A ++ DL T  PLA++ +SVSS  SDD  WSEE
Sbjct  614  IRTSLLAAVSHDLRT--PLASIKASVSSLRSDDVEWSEE  650


>ref|WP_026251764.1| histidine kinase [Streptomyces sp. BoleA5]
Length=847

 Score = 35.8 bits (81),  Expect = 5.2, Method: Composition-based stats.
 Identities = 19/39 (49%), Positives = 25/39 (64%), Gaps = 2/39 (5%)

Query  63   VEEELYALLASDLSTDNPLATLSSSVSSTGSDDDAWSEE  101
            +   L A ++ DL T  PLA + ++VSS  SDD AWSEE
Sbjct  614  IRTALLAAVSHDLRT--PLAAIKAAVSSLRSDDVAWSEE  650


>ref|WP_030211244.1| histidine kinase [Streptomyces bikiniensis]
Length=848

 Score = 35.8 bits (81),  Expect = 5.3, Method: Composition-based stats.
 Identities = 19/39 (49%), Positives = 25/39 (64%), Gaps = 2/39 (5%)

Query  63   VEEELYALLASDLSTDNPLATLSSSVSSTGSDDDAWSEE  101
            +   L A ++ DL T  PLA++ +SVSS  SDD  WSEE
Sbjct  614  IRTSLLAAVSHDLRT--PLASIKASVSSLRSDDVEWSEE  650


>ref|WP_030623816.1| MULTISPECIES: histidine kinase [Streptomyces]
Length=847

 Score = 35.4 bits (80),  Expect = 5.8, Method: Composition-based stats.
 Identities = 19/39 (49%), Positives = 25/39 (64%), Gaps = 2/39 (5%)

Query  63   VEEELYALLASDLSTDNPLATLSSSVSSTGSDDDAWSEE  101
            +   L A ++ DL T  PLA + ++VSS  SDD AWSEE
Sbjct  614  IRTALLAAVSHDLRT--PLAAIKAAVSSLRSDDVAWSEE  650


>ref|WP_028441142.1| MULTISPECIES: histidine kinase [Streptomyces]
 gb|KDQ69988.1| histidine kinase [Streptomyces sp. NTK 937]
Length=847

 Score = 35.4 bits (80),  Expect = 5.8, Method: Composition-based stats.
 Identities = 19/39 (49%), Positives = 25/39 (64%), Gaps = 2/39 (5%)

Query  63   VEEELYALLASDLSTDNPLATLSSSVSSTGSDDDAWSEE  101
            +   L A ++ DL T  PLA + ++VSS  SDD AWSEE
Sbjct  614  IRTALLAAVSHDLRT--PLAAIKAAVSSLRSDDVAWSEE  650


>ref|WP_014055421.1| histidine kinase [Streptomyces violaceusniger]
 gb|AEM81916.1| osmosensitive K+ channel signal transduction histidine kinase 
[Streptomyces violaceusniger Tu 4113]
Length=870

 Score = 35.4 bits (80),  Expect = 6.4, Method: Composition-based stats.
 Identities = 19/39 (49%), Positives = 25/39 (64%), Gaps = 2/39 (5%)

Query  63   VEEELYALLASDLSTDNPLATLSSSVSSTGSDDDAWSEE  101
            +   L A ++ DL T  PLA + ++VSS  SDD AWSEE
Sbjct  624  IRTALLAAVSHDLRT--PLAAIKAAVSSLRSDDVAWSEE  660


>ref|WP_003955552.1| histidine kinase [Streptomyces clavuligerus]
 gb|EDY50040.1| turgor pressure sensor [Streptomyces clavuligerus ATCC 27064]
 gb|EFG09852.1| Sensor protein [Streptomyces clavuligerus ATCC 27064]
Length=847

 Score = 35.0 bits (79),  Expect = 8.5, Method: Composition-based stats.
 Identities = 19/39 (49%), Positives = 25/39 (64%), Gaps = 2/39 (5%)

Query  63   VEEELYALLASDLSTDNPLATLSSSVSSTGSDDDAWSEE  101
            +   L A ++ DL T  PLA + ++VSS  SDD AWSEE
Sbjct  614  IRTALLAAVSHDLRT--PLAAIKAAVSSLRSDDVAWSEE  650


>ref|XP_002980315.1| hypothetical protein SELMODRAFT_444519 [Selaginella moellendorffii]
 gb|EFJ18575.1| hypothetical protein SELMODRAFT_444519 [Selaginella moellendorffii]
Length=2030

 Score = 35.0 bits (79),  Expect = 9.3, Method: Composition-based stats.
 Identities = 11/31 (35%), Positives = 22/31 (71%), Gaps = 0/31 (0%)

Query  18   KKTNTRNHFKKEKNEECIAKTKEVFEEYGIG  48
            K +   NHF ++++E+C+ + +EV E+Y +G
Sbjct  469  KFSGVENHFYRQQHEQCVKRAREVIEKYSLG  499


>ref|WP_038465608.1| hypothetical protein [Candidatus Paracaedibacter acanthamoebae]
 gb|AIK96852.1| hypothetical protein ID47_09070 [Candidatus Paracaedibacter acanthamoebae]
Length=867

 Score = 35.0 bits (79),  Expect = 9.4, Method: Compositional matrix adjust.
 Identities = 20/67 (30%), Positives = 34/67 (51%), Gaps = 0/67 (0%)

Query  16   LKKKTNTRNHFKKEKNEECIAKTKEVFEEYGIGRDSVDHTVNVFRHLVEEELYALLASDL  75
            L K TN  ++  + + EE I  T   FE  G GR+S   T     ++ +    +L ++D+
Sbjct  532  LPKVTNMPSNGNETEGEEAIQPTVSEFENTGSGRNSKQPTAAATENVDDSSEASLPSADI  591

Query  76   STDNPLA  82
            ++D P A
Sbjct  592  NSDTPTA  598


Lambda      K        H        a         alpha
   0.313    0.129    0.363    0.792     4.96 

Gapped
Lambda      K        H        a         alpha    sigma
   0.267   0.0410    0.140     1.90     42.6     43.6 

Effective search space used: 647961316667


  Database: nr
    Posted date:  Sep 23, 2015 12:05 AM
  Number of letters in database: 26,053,659,533
  Number of sequences in database:  71,551,133


Matrix: BLOSUM62
Gap Penalties: Existence: 11, Extension: 1
Neighboring words threshold: 11
Window for multiple hits: 40
```
